# Depth of Interbreed Difference in Postmortem Bovine Muscle Determined by CE-FT/MS and LC-FT/MS Metabolomics

**DOI:** 10.3390/metabo14050261

**Published:** 2024-05-01

**Authors:** Susumu Muroya, Yuta Horiuchi, Kazuki Iguchi, Takuma Higuchi, Shuji Sakamoto, Koichi Ojima, Kazutsugu Matsukawa

**Affiliations:** 1 Division of Animal Products Research, NARO Institute of Livestock and Grassland Science (NILGS), Tsukuba 305-0901, Ibaraki, Japan; koojima@affrc.go.jp; 2 Faculty of Veterinary Medicine, Kagoshima University, Korimoto 890-0065, Kagoshima, Japan; 3 Human Metabolome Technologies Inc., Tsuruoka 997-0052, Yamagata, Japan; 4 Science Research Center, Kochi University, Nankoku 783-8505, Kochi, Japan; thiguchi@kochi-u.ac.jp (T.H.); sshuji@kochi-u.ac.jp (S.S.); 5 Department of Agriculture and Marine Science, Kochi University, Nankoku 783-8502, Kochi, Japan

**Keywords:** beef, breed, Fourier transform mass spectrometry, metabolomics, mitochondria, postmortem aging, skeletal muscle

## Abstract

Japanese Brown (JBR) cattle have moderately marbled beef compared to the highly marbled beef of Japanese Black (JBL) cattle; however, their skeletal muscle properties remain poorly characterized. To unveil interbreed metabolic differences over the previous results, we explored the metabolome network changes before and after postmortem 7-day aging in the *trapezius* muscle of the two cattle breeds by employing a deep and high-coverage metabolomics approach. Using both capillary electrophoresis (CE) and ultra-high-performance liquid chromatography (UHPLC)–Fourier transform mass spectrometry (FT/MS), we detected 522 and 384 annotated peaks, respectively, across all muscle samples. The CE-based results showed that the cattle were clearly separated by breed and postmortem age in multivariate analyses. The metabolism related to glutathione, glycolysis, vitamin K, taurine, and arachidonic acid was enriched with differentially abundant metabolites in aged muscles, in addition to amino acid (AA) metabolisms. The LC-based results showed that the levels of bile-acid-related metabolites, such as tauroursodeoxycholic acid (TUDCA), were high in fresh JBR muscle and that acylcarnitines were enriched in aged JBR muscle, compared to JBL muscle. Postmortem aging resulted in an increase in fatty acids and a decrease in acylcarnitine in the muscles of both cattle breeds. In addition, metabolite set enrichment analysis revealed that JBR muscle was distinctive in metabolisms related to pyruvate, glycerolipid, cardiolipin, and mitochondrial energy production, whereas the metabolisms related to phosphatidylethanolamine, nucleotide triphosphate, and AAs were characteristic of JBL. This suggests that the interbreed differences in postmortem *trapezius* muscle are associated with carnitine/acylcarnitine transport, β-oxidation, tricarboxylic acid cycle, and mitochondrial membrane stability, in addition to energy substrate and AA metabolisms. These interbreed differences may characterize beef quality traits such as the flavor intensity and oxidative stability.

## 1. Introduction

The skeletal muscle structure and physiological function in farm animals are primarily determined by their genetic background, such as their breed, and spatiotemporal developmental programs, including growth and maturation in individual life [1]. The skeletal muscle metabolite composition is tightly coupled with the functional properties of muscle fibers, which are categorized into two major types: slow oxidative and fast glycolytic [2]. Each muscle fiber type has a distinct set of muscle contractile proteins, metabolic enzymes, and metabolites that are determined by genetic, developmental, and growth programs [1]. These components play crucial roles in the maintenance and activity of muscle cells, as demonstrated by the role of metabolites as energy substrates in the mitochondrial metabolism [3], such as glycolytic products and fatty acids (FAs). Therefore, the skeletal muscle—a mass of muscle fiber bundles—has distinct physiological and biochemical properties. 

Furthermore, muscle metabolites such as amino acids (AAs) and nucleotides contribute to the flavor development of meat in postmortem-aged muscle [4,5,6]. ATP degradation in postmortem muscle is particularly important in terms of meat quality because it leads to the temporal accumulation of inosine 5′-monophosphate (IMP), a major flavor compound in meat [7,8]. Farm animal muscles undergo further changes after slaughter in environments with controlled temperature and humidity. Through the process of farm animal production and meat storage, the AA, FA, and glycolytic product profiles also change [1]. Thus, muscle metabolites are essential in both living skeletal muscle tissues and postmortem-aged meat due to their potential major roles as regulatory molecules in living tissues and as meat quality determinants [9]. The metabolites contributing to meat quality depend on the breed of cattle and sheep [10,11,12]. 

Environment, diet, and exercise affect skeletal muscle development, growth, and maturation in farm animals, rodents, and humans [1]. Nevertheless, among the factors affecting the skeletal muscle characteristics in cattle, breed is a crucial factor influencing the final beef quality and production, as shown in the high marbling of Japanese Black (JBL) beef—the original Wagyu breed with highly marbled beef [13]. The phenotype of this breed is largely different from that of Japanese Brown (JBR) cattle, a minor population breed with moderately marbled beef and high growth performance [14]. The JBR breed comprises two pedigrees: 10% of animals comprise the Kochi and the remainder are the Kumamoto [15]. In recent years, JBR beef has been accepted by health-conscious consumers because of its balanced composition of lean muscle and intramuscular fat [16]. Despite the increasing demand for beef, little is known about its skeletal muscle properties, especially in terms of its metabolic differences from JBL cattle. 

To understand the mechanisms underlying metabolite changes in such processes and conditions of meat production, metabolomic approaches have been employed in studies on postmortem meat aging [6,17,18,19,20,21]. Recently, we determined the changes in the water-soluble metabolome profiles of JBR *longissimus* muscle beef during postmortem aging using capillary electrophoresis (CE)–time-of-flight mass spectrometry (CE-TOF/MS) [14]. The results demonstrated that metabolite changes over time were associated with the metabolism of pyrimidine, nicotinate, nicotinamide, purine, pyruvate, thiamine, amino sugars, and fatty acids, in addition to the tricarboxylic acid (TCA) cycle (namely citrate cycle) and pentose phosphate pathway. Most of these metabolisms and pathways were commonly observed in the metabolomic changes in JBL beef during postmortem aging [20]; however, JBR beef exhibited differences from JBL beef in the accumulation of choline, AAs, uridine monophosphate (UMP), IMP, fructose 1,6-diphosphate (FBP), and betaine [14]. 

The metabolomics results using CE-TOF/MS further suggested that the metabolism associated with nucleoside monophosphate, glutathione, and phospholipids during the postmortem aging of muscle differs between the two cattle breeds. Thus, most of the other postmortem metabolic pathways remain unknown, especially in terms of differences between cattle breeds and muscle types. To further explore interbreed differences, a more comprehensive metabolomics approach superior to CE-TOF/MS is required, as the range of CE-TOF/MS metabolomics was limited to water-soluble metabolites. 

In this study, we aimed to determine the interbreed differences in an expanded range of bovine muscle metabolisms, targeting slaughtered and postmortem 7-day-aged neck (*trapezius*) muscles. To address this, both CE and ultra-high-performance liquid chromatography (UHPLC-LC)–Fourier transform MS (FT/MS) were employed, which allowed us to perform high-performance metabolomics that can detect not only water-soluble and charged compounds, but also fewer polar compounds with high-resolution MS [9]. The metabolomic data of JBR (Kochi pedigree) and JBL cattle were subjected to bioinformatics analyses, including metabolite pathway analysis, to determine the differences in postmortem muscle aging. The present results revealed the depth of interbreed differences in the metabolome of aged beef and the metabolisms of *trapezius* muscle, including the involvement of mitochondrial stability and energy metabolism, which has never been approached to date.

## 2. Materials and Methods

### 2.1. Animals 

Two steers and one heifer each of the JBR cattle (the Kochi (Tosa) pedigree (JBRT)) and JBL cattle were fed and reared under the same feeding conditions in a Tajima farm (Nankoku, Kochi, Japan) until the age of 28 months. Individual diets were designed to satisfy their energy and other nutrient requirements based on the standard diet model of the Japanese Feeding Standard for Beef Cattle (JFSBC, 2008 ed.) [22]. The dietary composition was the same as that used in a previous JBL cattle experiment [14]. According to the JFSBC, the recommended levels of dry matter, neutral detergent fiber, total digestible nutrients, and crude protein in the diet are 87.1%, 33.5%, 75.8%, and 11.8%, respectively (all values are expressed as a percentage of total mixed rations). Since the difference in the metabolome profile between steers and heifers is not significant during postmortem aging [14], we considered that each group of breed could be designed as a mixture of steers and heifers. The neck (*trapezius*) muscle of the cattle was obtained immediately after slaughter. 

### 2.2. Muscle Samples

To analyze the effects of postmortem aging, *trapezius* muscle was dissected from the neck muscle within 1 h of slaughter. To avoid the contamination of the intramuscular fat, only the lean portion of the small muscle pieces was carefully collected from several locations in the muscle at time 0 postmortem (D0). After 7-day storage postmortem in a vacuumed bag at 2 °C, the small lean muscle portions were picked up. The mincing of muscle samples was avoided to minimize metabolite deterioration. All subsequent small pieces of lean muscle were collected and stored at −80 °C until use. 

### 2.3. CE-FT/MS Measurement

Samples for CE-FT/MS measurements were prepared as previously described [14]. In brief, the frozen muscle pieces (20.7–37.7 mg) were immediately soaked in 50% acetonitrile aqueous solution containing internal standards (H3304-1002, Human Metabolome Technologies, Inc. (HMT), Tsuruoka, Japan). After homogenization with zirconia beads (5 mmφ and 3 mmφ) at 1500 rpm, 4 °C for 60 s × 2 times using a beads shaker (Shake Master NEO, Bio Medical Science, Tokyo, Japan), the same amount of 50% acetonitrile/Milli-Q water was added to the mixture, and further homogenization was performed. The homogenate was then centrifuged at 2300× *g*, 4 °C for 5 min. Subsequently, the upper aqueous layer was centrifugally filtered through a Millipore 5-kDa cutoff filter (Ultrafree MC-PLHCC, HMT) at 9100× *g*, 4 °C for 120 min to remove macromolecules. The filtrate was evaporated to dryness under vacuum and reconstituted in 50 µL of Milli-Q water for CE-FT/MS analysis at HMT. Metabolome analysis was conducted according to HMT’s *ω Scan* package, using an Agilent CE system (Agilent Technologies, Waldbronn, Germany) combined with Q Exactive Plus MS apparatus (Thermo Fisher Scientific K.K., Tokyo, Japan). HMT Sheath Liquid H3301-1001 and H3302-1023 (HMT) were used as cation and anion electrophoresis buffers. The samples were injected at a pressure of 50 mbar and the CE voltage was set at 30 kV. Electrospray ionization–mass spectrometry (ESI-MS) was conducted in the positive and the negative ion modes with the capillary voltage set at 4000 and 3500 V for the cationic and anionic mode analyses, respectively. The spectrometer scanned from 60 to 900 *m*/*z* for the cation mode and from 70 to 1050 *m*/*z* for the anion mode [23].

### 2.4. UHPLC-FT/MS Measurement

For the samples used for UHPLC-FT/MS measurements, the frozen muscle pieces (20.5–32.5 mg) were immediately soaked in 1% formic acid/acetonitrile solution containing internal standards (H3304-1002, HMT). After three rounds of sample crushing for 120 s at 1500 rpm in a cold crasher, the crushing was repeated once after adding the 1% formic acid solution, followed by once after adding Milli-Q water. After centrifugation at 2300× *g* for 5 min at 4 °C, the upper layer of the solution was filtered through a 3 kDa cutoff filter (NANOCEP 3K OMEGA, PALL Corporation, MI, USA) at 9100× *g*, 4 °C for 120 min to remove macromolecules, and further filtered using a hybrid SPE phospholipid cartridge (Hybrid SPE—Phospholipid 30 mg/mL, SUPELCO, Tokyo, Japan) to remove phospholipids. The filtrate was lyophilized, suspended in 50% isopropanol aqueous solution, and applied to the LC-FT/MS analysis at HMT. Metabolome analysis was performed using a Vanquish Flex UHPLC System (Thermo Fisher Scientific) combined with an Orbitrap Exploris 240 MS system (Thermo Fisher Scientific). The systems were connected by an OctaDecylSilyl (ODS) column (2 mm *i.d.* × 50 mm, 2 μm), with mobile-phase buffers (A: H_2_O/0.1% HCOOH, B: Isopropanol: Acetonitrile: H_2_O (65:30:5)/0.1% HCOOH, 2 mM HCOONH_4_) for both the positive and negative modes. The flow rate was set at 0.3 mL/min. The gradient condition for the positive mode was B: 1% for 0–0.5 min, B: 1–100% for 0.5–13.5 min, and B: 100% for 13.5–20 min. For the negative mode, the gradient was B: 1% for 0–0.5 min, B: 1–100% for 0.5–13.5 min, and B: 100% for 13.5–20 min. The ESI-MS was conducted in the positive and the negative ion modes with the capillary voltage set at 2500 and 2300 V for the positive and negative mode analyses, respectively. The spectrometer scanned from 100 to 1500 *m*/*z*.

### 2.5. Data Analysis of MS Measurement Results

The raw data obtained by CE-FT/MS and UHPLC-FT/MS were processed using MasterHands software (ver. 2.19.0.2). The signal peaks corresponding to isotopomers, adduct ions, and other product ions of known metabolites were excluded, all signal peaks potentially corresponding to authentic compounds were extracted, and their migration times (MTs) or retention times (RTs) were normalized using those of the internal standards. Thereafter, alignment of the peaks was performed according to the *m*/*z* values and normalized MT or RT values. The detected peaks were annotated using HMT’s metabolite database. To compare the relative compound levels between time points and cattle breed types, the peak areas were normalized using the internal standard levels and sample weights. The absolute concentrations of some of the major compounds, such as glycolytic products, AAs, and ATP degradation products, were determined using commercially available standards. In the comparative analysis, the abundance of undetected compounds was set to zero. Raw MS data file conversion, peak picking, noise reduction, and data alignment for multiple samples were performed as previously described [14].

### 2.6. Statistical Analyses

Normalized relative content values were used for data analysis. MetaboAnalyst (https://www.metaboanalyst.ca/, accessed on 18 February 2024), a bioinformatics database, was used for principal component analysis (PCA), hierarchical cluster analysis (HCA), analysis of variance (ANOVA), post hoc multiple comparison tests, and metabolite set enrichment analysis (MSEA). For the statistical analysis, data were analyzed using one-way ANOVA with the breed or postmortem storage time as the fixed effects and the individual animal as the random effect, considering storage time as a within-subject factor. Data were considered significantly different at *p* < 0.05, and a different trend was considered if the *p*-value was <0.10. In PCA score plots, the confidence level of the ellipse is 95%.

## 3. Results

### 3.1. Interbreed Differences in the Neck Muscle Metabolome between JBR and JBL Cattle

#### 3.1.1. Overview of CE-FT/MS Metabolomics Results

In the metabolomics of this study, 522 and 384 annotated peaks were detected using CE-FT/MS and LC-FT/MS, respectively, across all muscle samples (Tables S1 and S2). These compounds included 794 unique compounds, of which at least 10 metabolites were annotated in common between CE-FT/MS and LC-FT/MS (decanoic acid, *N*-hexanoylglycine and riboflavin in CE-FT/MS, and cholic acid, glycocholic acid, myristic acid, prostaglandin E2, prostaglandin F2α and taurocholic acid in LC-FT/MS). 

**Figure 1:**
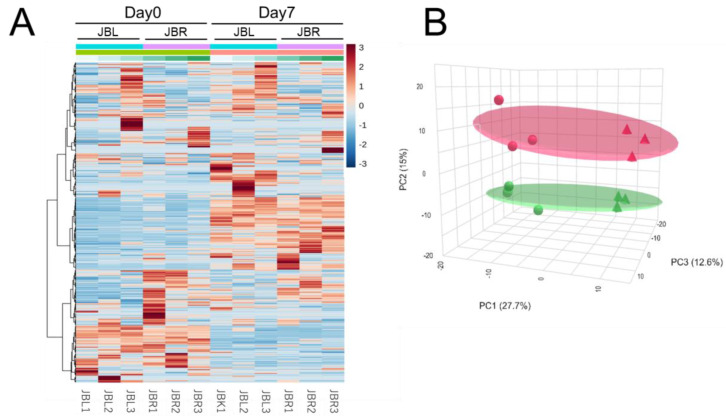
Hierarchical clustering analysis (**A**) and principal component analysis (PCA) (**B**) results using CE-FT/MS metabolomics profiles in the postmortem *trapezius* muscle of Japanese Brown (JBR; JBR1-3) and Japanese Black (JBL; JBL1-3) cattle. The muscle samples at a specific time point (day 0 and 7) were allocated to each breed. (**A**) In the heatmap, the row displays the metabolite, and the column represents the sample. Metabolites with relatively low and high levels are displayed in light blue and brown, respectively. The brightness of each color corresponds to the magnitude of the difference when compared to the average value. (**B**) In the PCA score plot, the muscle samples of day 0 (circle) and day 7 (triangle) are indicated for JBR (green) and JBL (red) cattle.

To determine the differences in the *trapezius* muscle metabolism and related metabolites between the two cattle breeds, we first conducted metabolomics using CE-FT/MS, followed by multivariate PCA and HCA data analyses, and bioinformatic MSEA analysis. In the HCA and PCA results, the muscle samples of the cattle before and after postmortem aging clustered depending on the aging time and cattle breed (Figure 1). Muscle metabolites were distributed into several categories with different patterns of association with age and breed in the HCA heatmap (Figure 1A). The muscle samples used in this study were separated by aging time points (PC1) and the two breeds (PC2), with PC1 and PC2 proportions of 27.7% and 15.0%, respectively (Figure 1B). These results demonstrated that the water-soluble metabolite composition was clearly different between the JBR and JBL breeds. Although the proportion of PC3 was 12.6%, the biological meaning of PC3 could not be determined.

#### 3.1.2. Differences in CE-FT/MS Metabolome Profiles between Cattle Breeds

**Figure 2:**
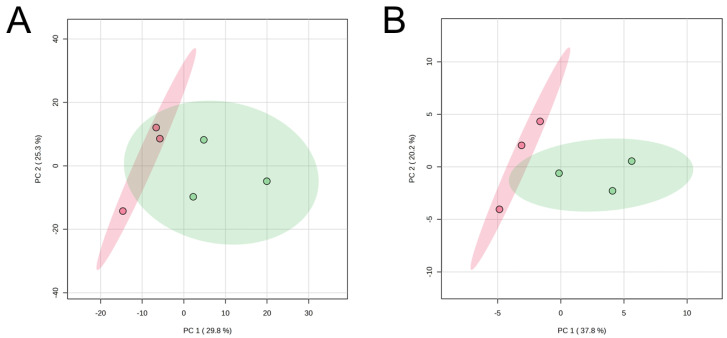
PCA score plots of CE-FT/MS metabolomics data of the *trapezius* muscle of JBR (in green) and JBL (in red) cattle on day 0 (**A**) and day 7 (**B**).

The PCA of the CE-based metabolomics using fresh (day 0) or aged (day 7) muscle samples revealed that the JBR and JBL muscle samples were not fully distinguished even by PC1 (Figure 2). Due to a lack of significant discrimination in PCA, we compared the metabolite levels using univariate statistical analysis, rather than further multivariate analysis, to simply extract meaningful metabolites. 

**Figure 3:**
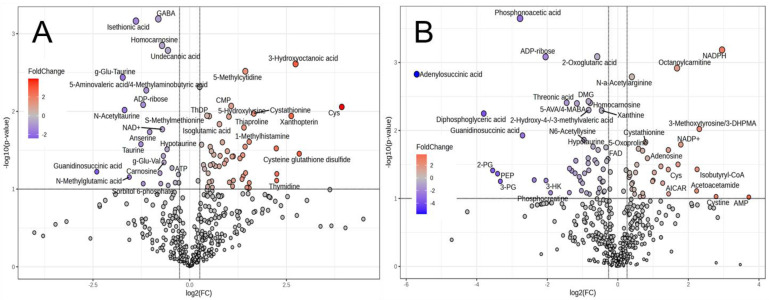
Volcano plot of CE-FT/MS metabolomics results of the *trapezius* muscle of JBR and JBL cattle on day 0 (**A**) and day 7 (**B**). The metabolites highly contributing to JBR and JBL (fold change > 1.5, *p* < 0.10) are indicated.

The JBR muscle on day 0 was characterized as having high levels of 3-hydroxyoctanoic acid, 5-methylcytidine, CMP, cysteine and cystathionine, and on day 7 was characterized as having an abundance of cysteine, isobutyryl-CoA, NADPH, octanoylcarnitine and *N*-α-acetylarginine (Figure 3). In JBL, GABA, isethionic acid, undecanoic acid, homocarnosine, γ-Glu-taurine, and ADP-ribose characterized the muscle, whereas on day 7, phosphonoacetic acid, ADP-ribose, 2-oxoglutarate (2OG), adenylosuccinic acid, *N*,*N*-dimethylglycine (DMG), threonic acid, and xanthine characterized the muscle. The significantly different metabolites are listed in Table S3.

**Figure 4:**
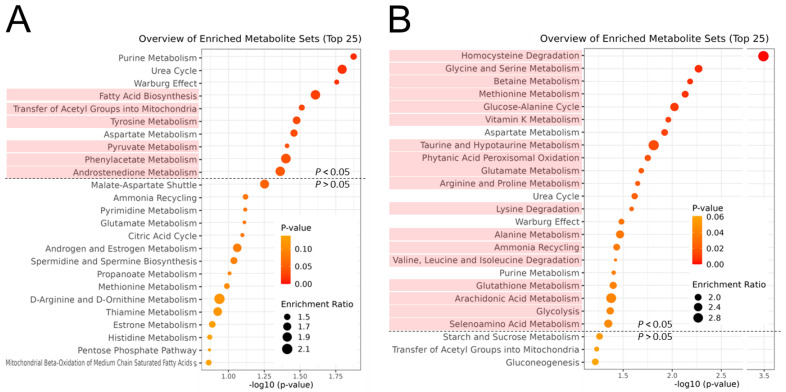
MSEA results for the metabolomic difference between JBR and JBL on day 0 (**A**) and 7 (**B**) postmortem. Enrichment ratio is computed by (observed hits)/(expected hits). Different metabolisms between the days at *p* < 0.05 are indicated in light red.

We performed MSEA to investigate the relevant biological events contributing to interbreed differences. Of the top 25 different metabolic pathways between the cattle breeds, the purine metabolism, urea cycle, Warburg effect, and aspartate metabolism were identified as significant biochemical events in both day 0 and day 7 muscles (Figure 4). FA biosynthesis, the transfer of acetyl groups into mitochondria, and the metabolisms related to tyrosine, pyruvate, phenylacetate, and androstenedione were extracted as highly different metabolic pathways between the breeds in day 0 muscle. Homocysteine degradation, the glucose–alanine cycle, phytanic acid peroxisomal oxidation, lysine degradation, glycolysis, and the metabolisms related to various AAs, vitamin K, taurine, glutathione, arachidonic acid, and selenoamino acid were significantly different on day 7.

The metabolisms extracted from day 0 were primarily characterized by higher levels of TPP in JBR cattle and higher levels of AMP in JBL cattle (Table 1). In contrast, the metabolites extracted from day 7 in JBR cattle were mainly characterized by higher levels of GSSG, NADPH, and TPP, whereas those in JBL cattle were represented by higher levels of 2OG. Based on the results, the JBR and JBL muscles on day 7 were distinguished by products of the NADPH-based redox metabolism, AA catabolism, glycolysis, and TCA cycle.

#### 3.1.3. Differences in LC-FT/MS Metabolome Profiles between Cattle Breeds

**Figure 5:**
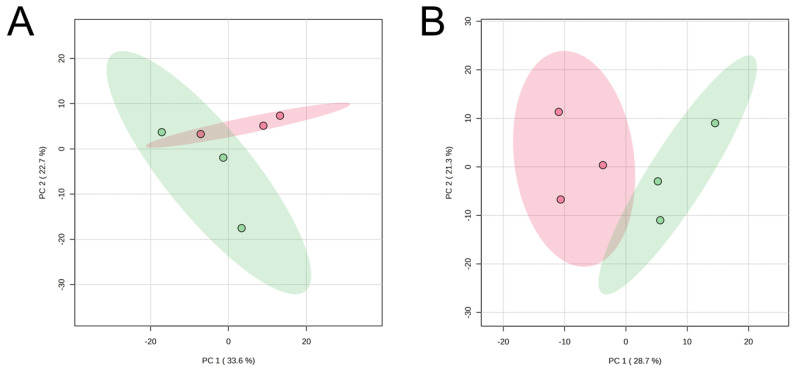
PCA score plots of LC-FT/MS metabolomics data of the *trapezius* muscle of JBR (in green) and JBL (in red) cattle on day 0 (**A**) and day 7 (**B**).

**Figure 6:**
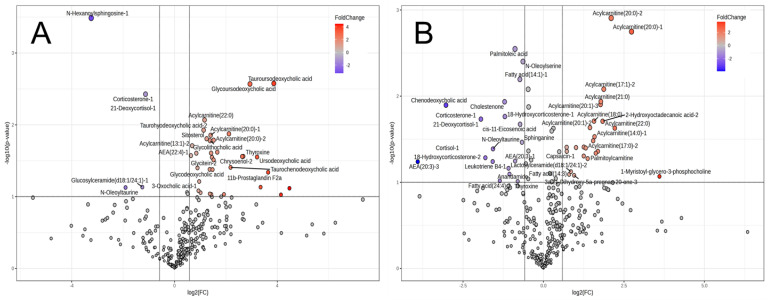
Volcano plot of LC-FT/MS metabolomics results of the *trapezius* muscle of JBR and JBL cattle on day 0 (**A**) and day 7 (**B**). The metabolites highly contributing to JBR and JBL (fold change > 1.5, *p* < 0.10) are indicated.

LC-FT/MS metabolomics was conducted to further explain the metabolomic range mainly targeting long-chain FAs (LCFAs). The PCA results revealed that the JBR and JBL muscle samples separated on day 7 but not on day 0, owing to breed differences (Figure 5). The LC-based metabolomics revealed that the differences in metabolite distribution between the two cattle changed as the muscle aged postmortem (Figure 6).

Based on the PC1 loading of the LC-based profiles on day 7, the JBR muscle was characterized as having 3-hydroxydodecanoic acid, cholesterol/lathosterol, 5α-pregnane-3,20-dione, γ-tocopherol, and sphingosine (d18:1) with highly positive values, whereas the JBL muscle was characterized as having *N*-oleoylserine, palmitoleic acid, fatty acid (14:1), *cis*-11-eicosenoic acid, and cholestenone, with highly negative values. 

On day 0, 39 differentially expressed metabolites were identified, which increased to 51 by day 7. The composition of different metabolites was altered during the postmortem period (Figure 6). Notably, bile-acid-related metabolites such as tauroursodeoxycholic acid (TUDCA), glycoursodeoxycholic acid (GUDCA), and taurohyodeoxycholic acid (THDCA) were more abundant, in addition to thyroxine and the acylcarnitines (13:1), (20:0), and (22:0) (Figure 6A); however, *N*-hexanoylsphingosine and corticosterone/21-deoxycortisol were less abundant in the JBR muscle than in the JBL muscle on day 0 (Figure 6A). On day 7, contrasting results showed that many long-chain acylcarnitines were more abundant in JBR than in JBL, whereas FAs such as oleic acid, palmitoleic acid, and *cis*-11-eicosenoic acid were abundant in JBL (Figure 6B, Table S4). In JBR muscle, the levels of taurine, hypotaurine, and 5-glutamyltaurine were lower, but the glycolic acid level was higher than in JBL muscle according to the CE-FT/MS results (*p* < 0.10). The metabolites with significant interbreed difference are listed in Table S4.

### 3.2. Postmortem Changes in the Neck Muscle Metabolome of JBR and JBL Cattle

#### 3.2.1. Changes in CE-FT/MS Metabolomic Profiles 

**Figure 7:**
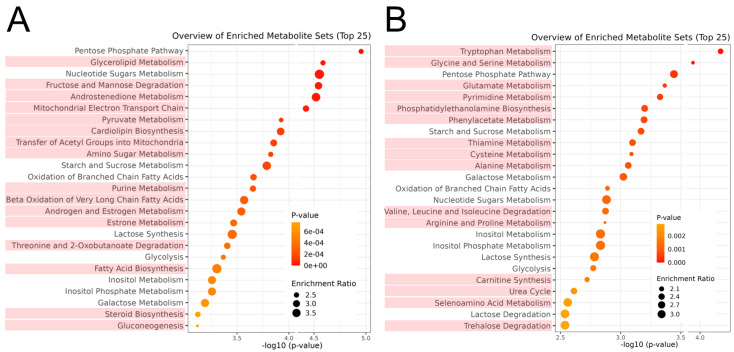
Result of MSEA for metabolomic differences between days 0 and 7 postmortem in JBR (**A**) and JBL (**B**) cattle. Enrichment ratio is computed by (observed hits)/(expected hits). The different metabolisms between the two breeds are indicated in light red.

Previously, we demonstrated changes in the metabolomic profile of the *longissimus* muscle of JBR and JBL cattle [14,20]. Numerous metabolites are associated with diverse muscle metabolisms, such as purine, pyrimidine, nicotinamide, pyruvate, thiamine, pentose phosphate, AA, FA metabolisms, and β-oxidation. Using high-resolution CE-FT/MS metabolomics, we analyzed the metabolomic changes in a slow and oxidative *trapezius* muscle during postmortem aging. As illustrated in Figure 1, the muscle samples collected on days 0 and 7 are clearly distinguishable in both cattle breeds. To determine the postmortem metabolism, MSEA was performed to determine the metabolomic profiles during the postmortem aging of the neck muscle in both cattle. Among the top 25 metabolic pathways extracted in MSEA, the pentose phosphate pathway, nucleotide sugar metabolism, starch and sucrose metabolism, oxidation of branched chain FAs, lactose synthesis, glycolysis, and inositol metabolism showed alteration during postmortem storage in both cattle breeds (Figure 7).

Notably, the metabolism that exhibited marked postmortem changes differed between the two breeds. In JBR cattle, the metabolism related to glycerolipids, sugars, the mitochondrial transport of FAs, pyruvate, cardiolipin, β-oxidation, and purines was highlighted. Meanwhile, the metabolisms of various types of AAs, pyrimidines, PEA, phenylacetate, thiamine, carnitine, and selenoamino acids highly contributed to the postmortem aging of the muscle in JBL cattle (Figure 7). The markedly changed metabolisms in JBL were enriched with AAs, including the catabolic metabolism, whereas mitochondrial metabolisms were characteristic of JBR. The metabolites significantly extracted from JBR cattle were mainly characterized by higher levels of ATP, NAD, TPP, and NADPH on day 0 and higher levels of acetyl-CoA and NADH on day 7 (Table 2). In addition, the significant metabolism in JBL cattle was characterized by a postmortem decrease in ATP and 2OG.

#### 3.2.2. Changes in LC-FT/MS Metabolomic Profiles 

**Figure 8:**
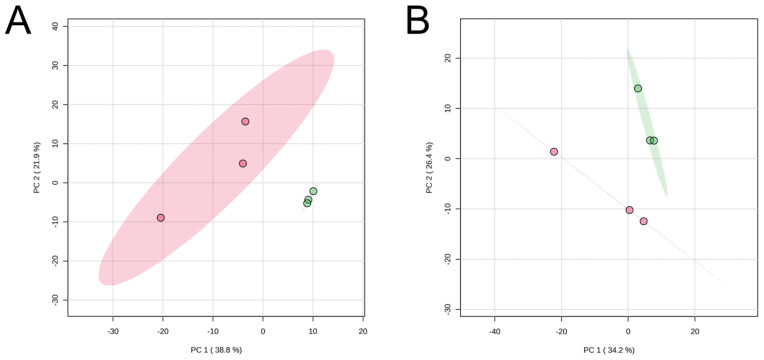
PCA score plots of LC-FT/MS metabolomics data of the *trapezius* muscle on day 0 (in red) and day 7 (in green) in JBR (**A**) and JBL (**B**) cattle.

**Figure 9:**
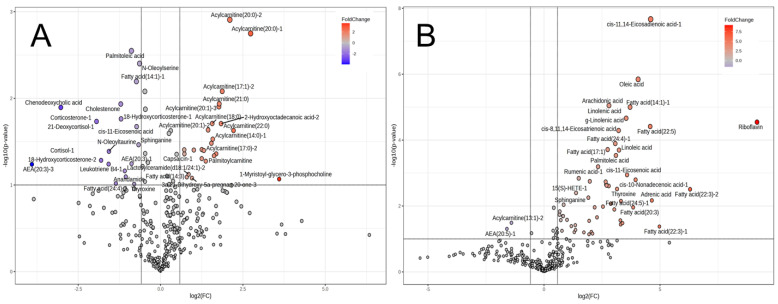
Volcano plot of LC-FT/MS metabolomics results of the *trapezius* muscle of JBR (**A**) and JBL (**B**) cattle. The metabolites highly contributing to day 0 and day 7 (fold change > 1.5, *p* < 0.10) are indicated.

The LC-based metabolomic profiles of JBR and JBL muscles were clearly different before and after postmortem 7-day aging (Figure 8 and Figure 9). The muscle samples in the PCA score plots were well distinguished into day 0 and day 7 groups by PC1 in JBR cattle but not by PC1 alone in JBL cattle (Figure 7B).

The JBR muscle was abundant in palmitoleic acid, FA (14:1), chenodeoxycholic acid (CDCA), *cis*-11-eicosenoic acid, and arachidonoylethanolamides (AEAs) on day 0, and in various acylcarnitines, 2-hydroxyoctadecanoic acid, and palmitoylcarnitine on day 7 (Figure 9A). In contrast, a few metabolites were specifically abundant in the muscles of JBL cattle on day 0 (Figure 9B). On day 7, LCFAs including oleic acid and linoleic acid, as well as riboflavin, accumulated in the JBL muscle (Figure 9B), which was in line with the representative metabolites with high PC1 loading.

Thus, CE-and LC-based metabolomics results revealed that acylcarnitines and carnitine increased during the postmortem aging of *trapezius* muscle in JBR muscle, which is not true for JBL cattle (Table 1, Figure 9). Sugar-related metabolisms, such as pentose phosphate, nucleotide sugar, and galactose, are also representative metabolic pathways in the postmortem muscles of both JBR and JBL cattle (Figure 7). The metabolism related to mitochondrial β-oxidation and membrane components such as cardiolipin was representative in JBR, while the phenylacetate, PEA, BCAA, and thiamine metabolisms were featured in JBL (Table 1). 

## 4. Discussion

The postmortem aging of animal skeletal muscle causes the catabolism of proteins, nucleotides, lipids, and various metabolites such as AAs [1]. Protein degradation has been particularly well described in postmortem meat aging [24,25,26], which gives rise to AAs in aged pork [6] and beef [14,20]. In the *longissimus* muscle of both JBR and JBL cattle, most of the postmortem AA generation and metabolite changes progress in a similar manner to previous CE-TOF/MS results. However, slight differences were observed between the two beefs [14], which raised the possibility of further interbreed differences. 

Herein, we employed highly encompassing CE-FT/MS and LC-FT/MS metabolomics to acquire more information in the present study than in previous studies. This approach allowed us to obtain a high-coverage metabolome profile and explore further details of the postmortem muscle metabolism, which revealed marked differences in postmortem *trapezius* muscle aging between the two cattle breeds. Owing to the higher number of detected compounds, the muscle samples tested in this study were more clearly separated by the cattle breed and postmortem aging time than those in the previous study [14] (as shown in the PCA and HCA results). Both cattle samples were distinguished before and after postmortem aging; however, the characteristic metabolites before and after aging differed between JBR and JBL cattle. The abundance of NADPH, octanoylcarnitine, and *N*-α-acetylarginine characterized JBR muscle on day 7 (Figure 3). These interbreed differences in metabolite groups indicate that the postmortem skeletal muscle metabolism differs. The metabolisms related to several AAs, glutathione, nicotinamide, purine, vitamin B6, spermidine, and betaine were previously identified as different metabolic pathways in postmortem *longissimus* muscle between cattle breeds [14]. These differences in muscle metabolites potentially affect the distinct meat quality of each breed, as previously shown in cattle and sheep [10,11,12]. 

Intriguingly, as shown in Figure 4 and Table 1, the present study has featured interbreed-different metabolisms relevant to FA, tyrosine, pyruvate, phenylacetate, and androstenedione in the *trapezius* muscle immediately after slaughter. In addition, the metabolism related to homocysteine degradation, the glucose–alanine cycle, phytanic acid peroxisomal oxidation, lysine degradation, glycolysis, and the metabolisms related to various AAs, vitamin K, taurine, glutathione, arachidonic acid, and selenoamino acids were markedly different in the postmortem muscle on day 7 between the two breeds. Furthermore, the acylcarnitine metabolism was one of the metabolic pathways that differed the most between the two cattle breeds in the LC-FT/MS analysis. On day 7, the levels of various long-chain acylcarnitines were higher in the JBR muscle than in the JBL muscle (Figure 6). Changes in the acylcarnitine levels during postmortem muscle aging have been previously observed in cattle and sheep [18,27], which is consistent with our results. 

**Figure 10:**
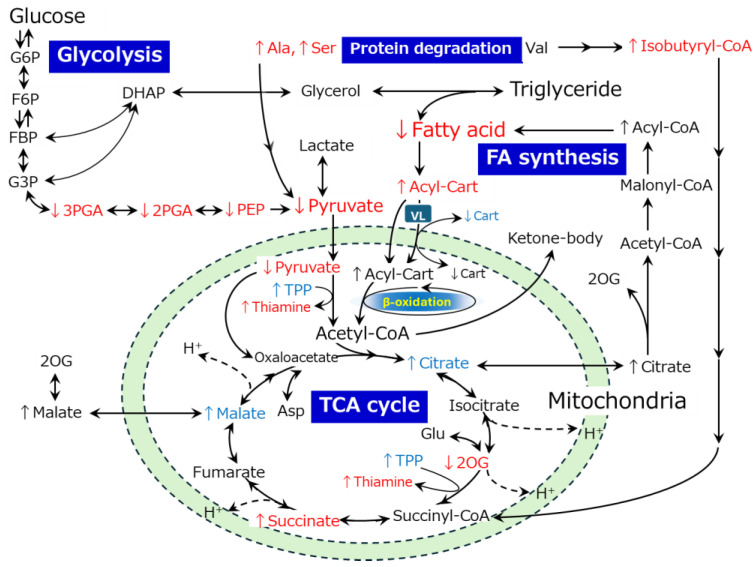
Hypothetical scheme of interbreed differences in the postmortem metabolism of the *trapezius* muscle between JBR and JBL cattle. The metabolites that differed between the JBR and JBL muscles on day 0 and day 7 are indicated in blue and red, respectively. ↑ and ↓ indicate a high and low level in JBR muscle compared to JBL muscle, respectively. VL; long and very long-chain FAs (VLCFAs), Cart; carnitine.

Compared to JBL muscle, a higher octanoylcarnitine level in JBR muscle (Figure 3) could be due to the low mitochondrial utilization of FA toward energy production through β-oxidation in the muscle (Figure 10). This is linked with the higher acylcarnitine levels in JBR muscle. The difference in mitochondrial stability suggested by postmortem changes in the membrane metabolisms could affect the distinct activity of carnitine palmitoyltransferases (CPTs) in the bovine muscles. Actually, changes in the CE-based metabolomic profile highlighted that the β-oxidation of very long FAs is one of the representative postmortem metabolisms in JBR (Figure 7A), with the characteristic decrease in octanoate, decanoate, and dodecanoate, and increase in acetyl-CoA and carnitine (Table 2). These interbreed differences in the acylcarnitine and LCFA metabolisms were likely due to the low activity of CPT-I and CPT-II, which catalyze LCFA transport from the cytoplasm into the mitochondrial matrix in muscle cells [28] in JBR muscle. This could be followed by the low activity of β-oxidation but not by that of the TCA cycle [29] in JBR.

In addition, the increase in acetyl-CoA in JBR muscle was likely due to the high glycolytic activity, but not due to FA mobilization, as suggested by the higher mobilization of glycolytic products (3PGA, 2PGA, PEP, and pyruvate; Table 2). The significant increase in acetyl-CoA and carnitine (Table 2) and decrease in the acylcarnitines (13:1), (20:0), and (22:0) (Figure 6A) during postmortem aging suggested that acylcarnitine biosynthesis was higher in JBR, whereas LCFA preparation was higher in JBL than in the counterpart breed. This does not contradict the abundance of citrate and malate on day 0 and succinate on day 7 in JBR muscle (Table 1), suggesting that mobilization of energy substrates were needed for the TCA cycle more in JBR muscle than in JBL muscle during postmortem aging (Figure 9). The significant increase in NADH in JBR muscle and high levels of energy products in the TCA cycle also supported this hypothesis (Table 2). As the result of a high mitochondrial activity before slaughter, molecular environment might become oxidative and make mitochondria unstable in JBR. Notably, acylcarnitines increased in JBR muscle but not in JBL muscle in the postmortem period; LCFAs increased in JBL muscle instead (Figure 9). This indicates that LCFAs did not undergo β-oxidation even if transported to the mitochondria in JBL muscle, whereas LCFAs were not transported to the mitochondria in JBR muscle (Figure 9). 

The levels of the glycolytic products 3PGA, 2PGA, and PEP, in addition to pyruvate, were lower in JBR muscle than in JBL muscle (Table 1), suggesting that the glycolytic activity was higher. In contrast, considering the acetyl-CoA accumulation on day 7 in JBR muscle (Table 1), the TCA cycle activity might be lower than in JBL muscle. Meanwhile, the higher level of TPP in JBR muscle on day 0 (Table 1) could play an essential role in the activation of pyruvate dehydrogenase and 2-oxoglutarate dehydrogenase to drive the TCA cycle, as shown by the low 2OG and high succinate levels in JBR muscle (Table 1). Furthermore, it is also likely that glucose and FAs were mobilized to the TCA cycle for energy production in the postmortem muscle of both beef cattle. Nevertheless, acetyl group transfer into mitochondria on day 0 and glycolysis and α-oxidation (phytanic acid peroxisomal oxidation) by day 7 occurred in JBR muscle, with higher metabolic activity than in JBL muscle (Figure 10), as shown in the MSEA results (Figure 4). 

Although the oxidation of FAs was over-representative of the postmortem metabolism in both JBR and JBL muscle (Figure 7), several AAs were highly degraded in JBL muscle compared to JBR muscle (Figure 4). The levels of glutamate, glutamine, and its products PGlu and 2OG differed between the two cattle breeds on day 0 and/or day 7 (Figure 4 and Table 1). In particular, the high isobutyryl-CoA level in the *trapezius* muscle of JBR (Figure 3) may have resulted from low valine generation by low protein degradation, in addition to impaired mitochondrial activity in aged beef. This trend is also observed in *longissimus* muscle, a faster type of muscle that differs from the *trapezius* muscle [14], implying that postmortem protein degradation is higher in JBL muscle than in JBR muscle, regardless of whether the muscle type is fast or slow. 

Furthermore, the metabolism related to membrane phospholipids such as glycerate was different between the cattle breeds and before and after postmortem muscle aging (Table 1). To date, the lipid postmortem changes in sheep [27,30] and beef [31] have been analyzed by a lipidomic approach, which provides evidence for the degradation of phospholipids, including mitochondrial phospholipids [31]. Although few postmortem phospholipid metabolic pathways have been explained in previous studies, the participation of glycerolipids, PEA, and cardiolipin metabolisms was shown in the present study. In JBR muscle, the levels of metabolites associated with phospholipid metabolism, such as CMP, CTP, and glycerate, were found to change during postmortem aging (Table 2). Likewise, in the postmortem JBL muscle, PEA biosynthesis was extracted as a significantly altered metabolism from the CE-based metabolomic profiles (Figure 7, Table 2). These postmortem changes and interbreed differences could contribute to a diverse meat quality, owing to the role of phospholipids in the generation of volatile compounds in meat [30].

In particular, the cardiolipin metabolism was extracted from the CE-based profile of JBR muscle (Table 1) because of the changes in related metabolites during postmortem aging [31]. In the bovine *psoas major* muscle, cardiolipin decreases during postmortem aging. Cardiolipin (a mitochondrial-membrane-specific phospholipid) has been demonstrated to interact with the channel core of the mitochondrial Ca^2+^ uniporter (MCU) complex [32]. The reduction in MCU levels and specific activity of cardiolipin-deficient yeast indicate that cardiolipin is essential for MCU stability and the regulation of mitochondrial Ca^2+^ signaling [33]. Accordingly, the changes in the cardiolipin metabolism (as deduced from its degradation) suggest that mitochondrial collapse and inactivation occurred during postmortem muscle aging, after which cardiolipin was released from the mitochondrial membrane and degraded, particularly in the JBR muscle. The symptoms of mitochondrial dysfunction have also been observed to be a decreased muscle oxygen consumption, mitochondrial protein content, and antioxidant capacity [34]. Parallel postmortem glycolysis and NAPDH depletion likely promote mitochondria dysregulation via pH decline and an increased cellular oxidative environment. 

Arachidonic acid is an essential ω-6 fatty acid present in a form of membrane phospholipid [35]. The arachidonic acid level was elevated in the postmortem muscles of both JBR and JBL tissues (Figure 9, Table S2), which coincided with a decrease in *cis*-11-eicosenoic acid, *cis*-11,14-eicosadienoic acid, and *cis*-8,11,14-eicosatrienoic acid in these tissues (Figure 9, Table S2). Interbreed differences in these eicosanoids may illustrate the different membrane stability, as suggested by the change in anandamide (AEA), an arachidonic-acid-related metabolite, from membranous phospholipids during postmortem muscle aging. Thus, the present results uncovered diverse postmortem releases and the subsequent degradation of membranous phospholipids from the plasma membrane, in addition to mitochondrial membrane. 

GUDCA, THDCA, and TUDCA were more abundant in JBR muscle than in JBL muscle on day 0 (Figure 6A), and decreased on day 7 postmortem in the muscle (Figure 6B). This may lead to potential differences or changes in the postmortem mitochondrial stability. The different TUDCA levels might be linked to higher levels of taurine and hypotaurine in JBL muscle than in JBR muscle (Table 1). The secondary bile acids TUDCA and GUDCA are the taurine and glycine conjugates of ursodeoxycholic acid (UDCA). Being generated as a primary bile acid in the liver from cholesterol, chenodeoxycholic acid (CDCA) is secreted into the large intestine [36]. UDCA is produced exclusively from CDCA by the microbiota, returns to the liver via the enterohepatic circulation and is conjugated with taurine to form TUDCA. The higher level of TUDCA in JBR muscle than that in JBL muscle may indicate an interbreed difference in circulating TUDCA levels. THDCA plays a cytoprotective role as a chemical chaperone in mitochondrial adaptation and membrane stability [36] by alleviating endoplasmic reticulum (ER) stress and stabilizing the unfolded protein response (UPR) [37], even in skeletal muscle cells [38,39]. Accordingly, different muscular THDCA levels at slaughter may result in interbreed differences in the mitochondrial stability and membrane component metabolism in the muscle. 

Although none of the meat quality traits were analyzed in this study, interbreed differences in beef metabolites potentially affect the meat quality traits. IMP, one of the most important flavor components, showed a higher level in the JBL *trapezius* muscle than in the JBR muscle on day 7 postmortem (*p* = 0.083) (Tables S1 and S3). Meanwhile, a previous study revealed that the IMP level in the JBR *longissimus* muscle is higher than in JBL muscle on day 14 postmortem [14]. Accordingly, even with modification by the muscle type effect, the interbreed difference in the IMP level may affect the beef flavor of each breed. Unlike the case of *longissimus* muscle, the AA levels were not lower in the JBR *trapezius* muscle than in the JBL muscle (Table S3) [14]. Accordingly, the contribution of AAs to the interbreed difference in beef flavor is likely limited in the *trapezius* muscle. In addition, given that mitochondrial activity is associated with the cellular redox condition, the interbreed difference in metabolite distribution may also affect lipid oxidation, the subsequently off-flavor of lipid peroxide and the meat color. The interbreed differences observed here could be utilized in future investigations evaluating the association with genetic background, to improve beef quality traits.

Metabolites related to oxidation stability such as glutathione and nicotinamide-related compounds modulate the cellular redox environment [40]. The accumulation of the oxidized form of these metabolites indicates a trend related to the oxidation of proteins and lipids [41,42], which also implies the deterioration of meat quality traits such as the water-holding capacity, meat color and flavor. In the present study, the levels of sulfur-containing metabolites, e.g., sulfur AAs (Cys, Table S3) [43], cystathionine and SLGT (Figure 3, Table 1), in skeletal muscle differed between cattle breeds and between aging times, suggesting that these redox-related metabolites participated in the cellular oxidative environment in the meat production process. 

A trend of higher FA accumulation in JBL muscle than in JBR muscle was possibly due to originally higher mitochondrial activity in the JBR *trapezius* muscle, as shown in differences in the levels of TCA cycle metabolites (Table 1), long-chain FAs, and acylcarnitines (Figure 6, Tables S2 and S4). Although mitochondrial oxidative phosphorylation (OXPHOS) terminates after death in the muscle due to oxygen depletion, oxidant metabolites were generated in the dysregulated redox condition [41], which could lead to lipid oxidation and the generation of reactive oxygen species (ROS) [44,45]. This may have led to high levels of oxidation products in the JBR muscle compared to the JBL muscle in this study. Furthermore, the present results indicated an interbreed difference in the degradation of membrane components, including mitochondrial ones (Table 2 and Table S2, Figure 4, Figure 6 and Figure 9), which could have led to differences in membrane lipid release and oxidation [31]. Such oxidation products may participate in the enhancement of oxidation and thereby color instability and the occurrence of an off-flavor in the meat [18,44]. 

## 5. Conclusion

The interbreed differences in bovine *trapezius* muscle were observed in the different distribution of AAs, FAs, nucleotides, and acylcarnitines in live skeletal muscle, but also on the postmortem muscle metabolisms. The metabolites of each breed generated during postmortem aging were diversified through complicated catabolism and oxidation process. The difference in metabolisms between the breeds, such as acylcarnitine transport, pyruvate metabolism, glycolysis, and the TCA cycle, could converge primarily to a distinct energy substrate mobilization in the muscle. Furthermore, based on differences in the unveiled muscle metabolites, the membranous phospholipid degradation was also likely different between JBR and JBL beef cattle. Thus, the interbreed differences in postmortem muscle metabolisms are profoundly linked to carnitine/acylcarnitine transport, energy supply and mitochondrial membrane stability, as well as AA metabolisms. These results contribute to a further understanding of the interbreed differences in beef quality, including flavor and oxidative stability.

## Figures and Tables

**Table 1:** Characteristic metabolisms contributing to interbreed differences in the *trapezius* muscle on day 0 and 7 postmortem.

Time Postmortem	Metabolism	Metabolites ^#^	
		Higher in JBR	Higher in JBL
Day 0	Fatty Acid Biosynthesis	Dodecanoic acid	Capric acid
	Transfer of Acetyl Groups into Mitochondria	Citrate, Malate, TPP	
	Tyrosine Metabolism	Asp, Tyramine	Vanylglycol
	Pyruvate Metabolism	Malate, TPP	AMP
	Phenylacetate Metabolism	Gln	AMP
Day 7	Homocysteine Degradation	Cystathionine, Ser	
	Betaine Metabolism	Adenosine	Betaine, DMG
	Vitamin K Metabolism	NADPH	
	Taurine and Hypotaurine Metabolism		Taurine, Hypotaurine, GLT
	Phytanic Acid Peroxisomal Oxidation	Isobutyryl-CoA, NADPH, Succinate, TPP	2OG
	Lysine Degradation	NADPH, TPP	2OG
	Valine, Leucine and Isoleucine Degradation	Isobutyryl-CoA, Succinate, TPP	2OG
	Glutathione Metabolism	GSSG, PGlu, NADPH, Ala	
	Arachidonic Acid Metabolism	GSSG, NADPH	
	Glycolysis		PEP, DPGA, 3PGA, Pyruvate
	Selenoamino Acid Metabolism	Adenosine, Ser, Ala	

^#^ The levels of the metabolites listed in Table 1 are higher in JBR or JBL than those in the counterpart breed at *p* ≤ 0.10.

**Table 2:** Representative metabolisms contributing to postmortem interbreed differences.

Cattle Breed	Metabolism	Metabolites *	
		Higher on Day 0	Higher on Day 7
JBR	Glycerolipid Metabolism	ATP, NAD, NADPH	Glycerate, NADH
	Mitochondrial Electron Transport Chain	ATP, NAD	NADH, Succinate
	Pyruvate Metabolism	ATP, GTP, NAD, TPP, NADPH, SLGT	NADH, Acetyl-CoA
	Cardiolipin Biosynthesis	CTP, NAD	CMP, NADH
	Transfer of Acetyl Groups into Mitochondria	ATP, Citrate, NAD, TPP, NADPH	Acetyl-CoA, NADH
	Purine Metabolism	Asp, ATP, cAMP, dGTP, GTP, NADPH	Adenosine, AICAR, AMP, Deoxyguanosine, Guanine, Guanosine, IMP, NADH, Xanthine, Xanthosine
	Beta Oxidation of Very Long Chain Fatty Acids	Decanoate, Dodecanoate, Octanoate	Acetyl-CoA, Carnitine
	Threonine and 2-Oxobutanoate Degradation	ATP, NAD, TTP	NADH
JBL	Pyrimidine Metabolism	ATP, Glu, PRPP, Thymine, TTP, UMP, Uracil, Uridine, UTP	dCMP
	Phosphatidylethanolamine Biosynthesis	ATP, Ethanolamine, Ser	
	Phenylacetate Metabolism	ATP, Gln	
	Thiamine Metabolism	ATP	TPP
	Valine, Leucine and Isoleucine Degradation	ATP, Isobutyryl-CoA, 2OG, Val	TPP, Ile
	Carnitine Synthesis	2OG, SAM, TMAB	
	Selenoamino Acid Metabolism	ATP, Ser	Ala

* The levels of metabolites listed in Table 2 are higher on day 0 or day 7 than in the counterpart time point at *p* ≤ 0.10.

## Data Availability

The original contributions presented in the study are included in the article/Supplementary Material, further inquiries can be directed to the corresponding author/s.

## Supplementary Materials

The following supporting information can be downloaded at: https://www.mdpi.com/article/10.3390/metabo14050261/s1, Table S1: Detected features and information on the identification of the annotated peaks in CE-FT/MS analysis, Table S2: Detected features and information on the identification of the annotated peaks in LC-FT/MS analysis, Table S3: List of metabolites that differed between cattle breeds at *p* < 0.10 in CE-FT/MS, Table S4: List of metabolites that differed between cattle breeds at *p* < 0.10 in LC-FT/MS.

## Abbreviation

ADP, adenosine diphosphate; AEA, anandamide; AICAR, 5-aminoimidazole-4-carboxamide ribonucleotide; Ala, alanine, AMP, adenosine monophosphate; Asp, aspartate; ATP, adenosine triphosphate; cAMP, cyclic AMP, CMP, cytidine monophosphate; CoA, coenzyme A; CTP, cytidine triphosphate; Cys, cysteine; DAMP, deoxyadenosine monophosphate; dCMP, deoxy CMP; dGTP, deoxy GTP; DHAP, dihydroxyacetone phosphate; DMG, dimethylglycine; DPGA, 2,3-Diphosphoglyceric acid; FBP, fructose 1,6-bisphosphate; F6P, fructose 6-phosphate; GABA, γ-aminobutyric acid; G6P, glucose 6-phosphate; G3P, glyceraldehyde 3-phosphate; Gln, glutamine; Glu, glutamate; GlcNAc6P, N-acetyl-glucosamine 6-phosphate; GLT, 5-glutamyl taurine; Gly, glycine; GSH, glutathione; GSSG, oxidized glutathione; His, histidine; HTrp, 5-hydroxytryptophan; IMP, inosine 5′-monophosphate, Lys, lysine; Met, methionine; MTA, 5′-methylthioadenosine; NADH, nicotinamide adenine dinucleotide; NAD, oxidized NADH; NADPH, NADH phosphate; NADP, oxidized NADPH; 2OG; 2-oxoglutarate, PEA, O-phosphoethanolamine; PEP, phosphoenolpyruvate; 3PGA, 3-Phosphoglyceric acid; PGlu, Pyroglutamic acid; PRPP, phosphoribosyl pyrophosphate; SAM, S-adenosylmethionine; SAHC, S-adenosylhomocysteine; Ser, serine; SLGT, S-lactoylglutathione; TMAB, 4-trimethylammoniobutanoic acid; TPP, thiamine pyrophosphate; Trp, tryptophan; TTP, thymidine triphosphate; Tyr, tyrosine; UDP, uridine 5′-diphosphate; UTP, uridine triphosphate; Val, valine.
